# Genomic diversity of *Salmonella* Typhimurium and its monophasic variant in pig and pork production in France

**DOI:** 10.1128/spectrum.00526-24

**Published:** 2024-11-08

**Authors:** Madeleine De Sousa Violante, Carole Feurer, Valérie Michel, Karol Romero, Ludovic Mallet, Michel-Yves Mistou, Sabrina Cadel-Six

**Affiliations:** 1 MaIAGE, INRAE, Université Paris-Saclay, Jouy-en-Josas, France; 2 ACTALIA, La Roche-sur-Foron, Haute-Savoie, France; 3 IFIP–Institut du Porc, Pôle Viandes et Charcuteries, Pacé, France; 4 Salmonella and Listeria Unit (SEL), ANSES, Laboratory for Food Safety, Maisons-Alfort, France; 5 Institut Universitaire du Cancer de Toulouse–Oncopole, Toulouse, Haute-Garonne, France; Health Canada, Ottawa, Canada

**Keywords:** *Salmonella*, Typhimurium, genomic, pig

## Abstract

*Salmonella* Typhimurium and its monophasic variant (*Salmonella* 4,[5],12:i:-) are among the most prevalent serovars worldwide. Even though these serovars have been the focus of many studies, their spread has not yet been investigated in French pig herds and slaughterhouses at a regional scale. Here, we characterized the genomic diversity of 188 *Salmonella* strains belonging to sequence type (ST) 19 and 34. These strains were isolated from pigs in metropolitan France between 2014 and 2019. Samples were collected from 10 regions, three of which together represent 75% of French pig production in 2020. To contextualize the French *Salmonella* genomes at a worldwide level, 193 ST 34 genomes from three continents and 14 countries were also included. This study revealed little diversity in ST 34 strains circulating in France, suggesting that one or two clones are spreading within pig herds and slaughterhouses. *In silico* virulence and antimicrobial resistance genes were investigated to understand the prevalence of these strains among farmed pigs and in the slaughterhouse environment. A comparison with ST 34 isolates from other countries highlighted the genomic specificity of the ST 34 monophasic variants in France, with some exceptions concerning isolates from bordering countries. This work provides new insights into the dynamics of *S*. Typhimurium and its monophasic variant sampled in French pig herds and slaughterhouses.

**IMPORTANCE:**

Salmonellosis is a leading cause of bacterial infection in humans and animals around the world. This study provides a snapshot of the genomic diversity of one of the most prevalent *Salmonella* serovars (*Salmonella* Typhimurium and *Salmonella* 4,[5],12:i:-) circulating on French pig farms between 2013 and 2021. We investigated the link between geographical and genomic diversity. The analyses revealed little diversity of the strains, suggesting that one or two clones are spreading within French pig herds. We also *in silico* screened genetic elements that could explain the prevalence of these strains among farmed pigs and in the slaughterhouse environment. Finally, the comparison with isolates from other countries highlighted the genomic specificity of these two French sequence type 34 clones. This work provides new insights into the dynamics of *S*. Typhimurium and *S*. 4,[5],12:i:- sampled from pig herds and slaughterhouses in France, thus laying the foundations for future analyses.

## INTRODUCTION

*Salmonella enterica* subsp. *enterica* serotype Typhimurium is a broad-host-range strain, isolated from humans, livestock, domestic fowl, rodents, and wild birds (1, 2). In the mid-1990s, a monophasic variant of Typhimurium (*Salmonella* 4,[5],12:i:-) first appeared in Europe (3). This serovar is characterized by its resistance to ampicillin, streptomycin, sulfonamides, and tetracycline (4), representing a major public health issue. Indeed, its incidence dramatically increased between 2005 and 2008, when it became one of the top three isolated serovars in human health, a position it still holds (5). *S*. 4,[5],12:i:-, together with *Salmonella* Enteritidis and *S*. Typhimurium represented 77.1% of the confirmed human cases reported in the EU in 2022 (6). Pork is a major vector of *Salmonella* (7–9) and thus is the most significant source of meat responsible for human transmission of *S*. 4,[5],12:i:- (10). Non-typhoidal *Salmonella* strains isolated from the pig and pork industries are primarily serovars *S*. 4,[5],12:i:- and *S*. Typhimurium (10, 11), which account for between 40% and 60% of all serovars found in pork between 2008 and 2020 in France (11).

In pork, some studies have focused on the evolution of *S*. 4,[5],12:i:- and *S*. Typhimurium in Europe or at the world level (12–14). At the country level, studies tend to focus on the overall diversity and antibiotic resistance (15–17) but do not give information on any genomic links between pig herds in different regions. However, this information sheds light on the geographical spread of other serovars in previous studies (18–21), which highlighted phylogeographic clades and adaptative advantages through antibiotic resistances profiles. There is, therefore, a need to investigate the diversity of these two serovars at the pig herd level and compare it with other countries to better understand any transfer of contamination.

In the present study, we analyzed the core genome phylogeny and the accessory genome [*Salmonella* pathogenicity island (SPI), virulence factors, and antibiotic resistance] of 188 *S*. Typhimurium and *S*. 4,[5],12:i:- strains belonging to multilocus sequencing typing (MLST) profiles sequence type (ST) 19 and ST 34. These strains were obtained from French farms and slaughterhouses between 2014 and 2019. The epidemiological data on the farms from which each sample came were collected to depict the overall geographical diversity of the ST 19 and ST 34 clones circulating in France. Finally, a panel of 193 genomes belonging to ST 34 from pigs reared in foreign countries was used to explore the diversity of French *S*. 4,[5],12:i:- clones at the international level.

## MATERIALS AND METHODS

### Selection of French strains

**Fig 1:**
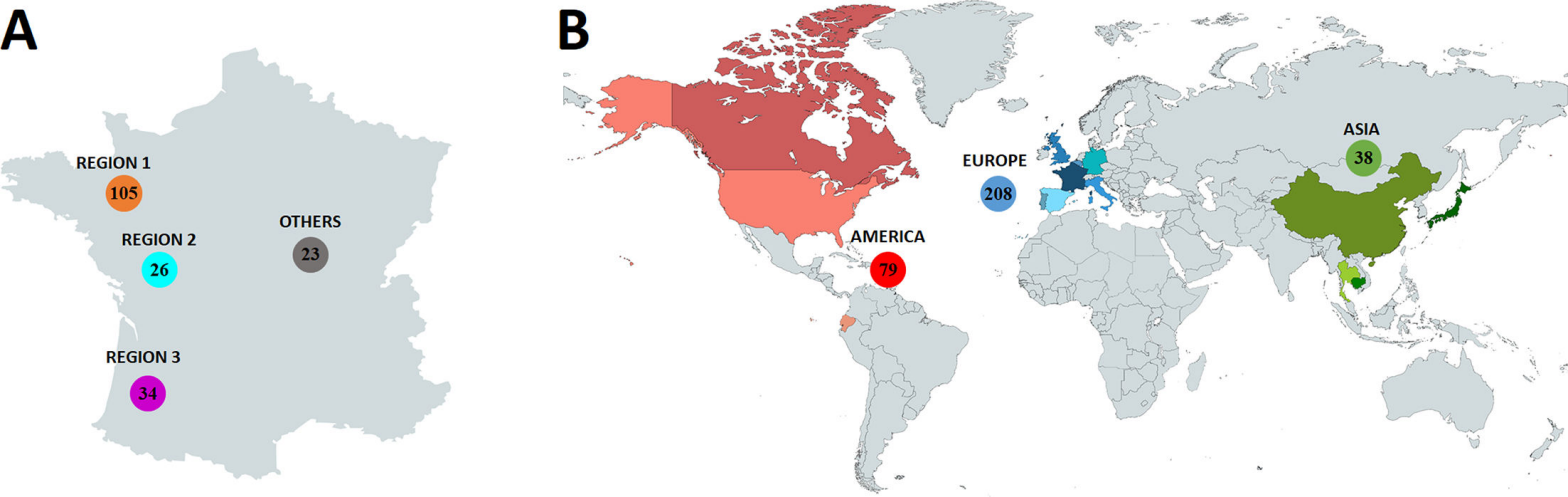
Illustration of the data set collection (generated using www.mapchart.net). (A) Samples collected in France by region. (B) Samples collected from France and Enterobase by continent.

To study the genomic diversity of *S*. Typhimurium and *S*. 4,[5],12:i:-, 188 strains with their epidemiological data [name of the pig breeding department (a department being a French administrative unit), sampling year, and source] were selected from the IFIP-Institut du Porc and ANSES collections (*Salmonella* Network collection of the Food Safety Laboratory in Maisons-Alfort and the National Reference Laboratory for Antibiotic Resistance collection in Fougères) (Table S1). After deduplication, all the strains collected during French regulatory monitoring plans between 2014 and 2019 were included. One of the goals of this monitoring plan was to sample strains that covered the national production of pigs. Samples were taken directly from breeding animals, fattening pigs, and feces on the farms (*n* = 21) and by swabbing pig carcasses and tongues at the slaughterhouse (*n* = 167) (Table S1, “French genomes” tab). For the strains isolated at the slaughterhouse, the department where each sampled pig had been bred was recorded. Among the 188 strains, 165 were isolated from the three French regions that are the most involved in pig production: Brittany, henceforth called “region 1” (*n* = 105 strains), which is in the north-west of France and accounts for 78% of French pig production (22); Pays-de-la-Loire, “region 2” (*n* = 26), in the mid-west; and Nouvelle-Aquitaine, “region 3” (*n* = 34), in the south-west, the latter two corresponding to the second and third most important pig production regions, respectively. Twenty-three more strains from other French regions were also chosen to geographically cover metropolitan France. The latter strains are later called “others” in the study. Among the 188 strains, 181 were identified as belonging to Typhimurium and 1.4,[5],12:i:- by glass slide agglutination, according to the White-Kauffmann-Le Minor scheme (23). The seven remaining strain serovars were predicted by SeqSero2 (24). The 188 strains selected (Fig. 1A) were sequenced using Illumina chemistry producing paired-end reads as described by Radomski et al. (25).

### Worldwide genome selection

Raw reads from foreign countries were retrieved from Enterobase (26). From 21,191 available samples (downloaded in August 2021), only ST 34 genomes isolated in the same time span as French genomes (from 2014 to 2019) with available Illumina raw reads were selected. Raw reads were filtered to select only swine sources whose country of origin was known. The first selection gave 964 samples, including 858 samples isolated in the USA. Further criteria were added to USA samples, such as the serotyping prediction from SeqSero2 and the geographical metadata available to downsize the data set to 51 samples. For the other countries, all 104 samples were retained. Thirty-eight genomes from the COMPARE project (12) were included in the final panel. Genomes were selected from Germany (*n* = 22), the UK (*n* = 6), and Italy (*n* = 10) from different pig sources but always in the same time span as the French strains. In total, 193 samples from foreign countries were selected (Fig. 1B; Table S1: “International genomes” and “ST 34 genome panel”).

### Sequencing, assembly, and quality assessment

Illumina paired-end raw reads were assembled using the ARTwork pipeline (27). Genomes with many assembled bases unaligned to the species reference or many InDels per 100 kb computed by QUAST (28) were discarded from the study. Also, samples with inter- or intra-genus contamination according to the default Confindr (29) parameters (samples with multiple genera found in the Mash screen step or more than two single nucleotide variants in ribosomal genes) were discarded from the study. Samples were serotyped *in silico* based on the assembled genomes using SeqSero2 (24), while the seven-gene Achtman scheme sequence type of each genome was computed by the MLST tseeman tool (https://github.com/tseemann/mlst) (Table S1).

### cgSNP caller and phylogenomic inference

Core genome analysis was performed by the iVARCall2 workflow (30), which detects single nucleotide polymorphisms (SNPs) and small InDels following the best practices proposed by the Genome Analysis ToolKit (31). Reads were mapped to the *Salmonella* Typhimurium LT2 (NCBI NC_003197.1) genome reference, and duplications were excluded before variant calling analysis via local *de novo* assembly of haplotypes in active regions. Recombination tracks were identified using ClonalFrameML (32) with the following parameters set to true: -em, -guess_initial_m, -use_incompatible_sites, -reconstruct_invariant_sites, -output_filtered. The parameter -emsim was set to 20, and other parameters were kept at their default values. The phylogenomic tree was inferred by IQTREE (33), used with TEST model selection on alignments including and excluding variants from homologous recombination detected previously, and robustness was tested with -alrt 1000. Phylogenomic tree plots were generated using iTOL (34).

### Accessory genome analysis

The 188 genomes from France were screened for the presence/absence of genes mediating resistance and virulence using ABRICATE (https://github.com/tseemann/abricate) with a threshold set at 90% identity over 3/5 of the length of the genomic region. ABRICATE was used against the VFDB database (35), ResFinder (36), and SPIFinder (37).

## RESULTS

### Depicting the geographical diversity of *S*. Typhimurium and *S*. 4,[5],12:i:- in France

**Fig 2:**
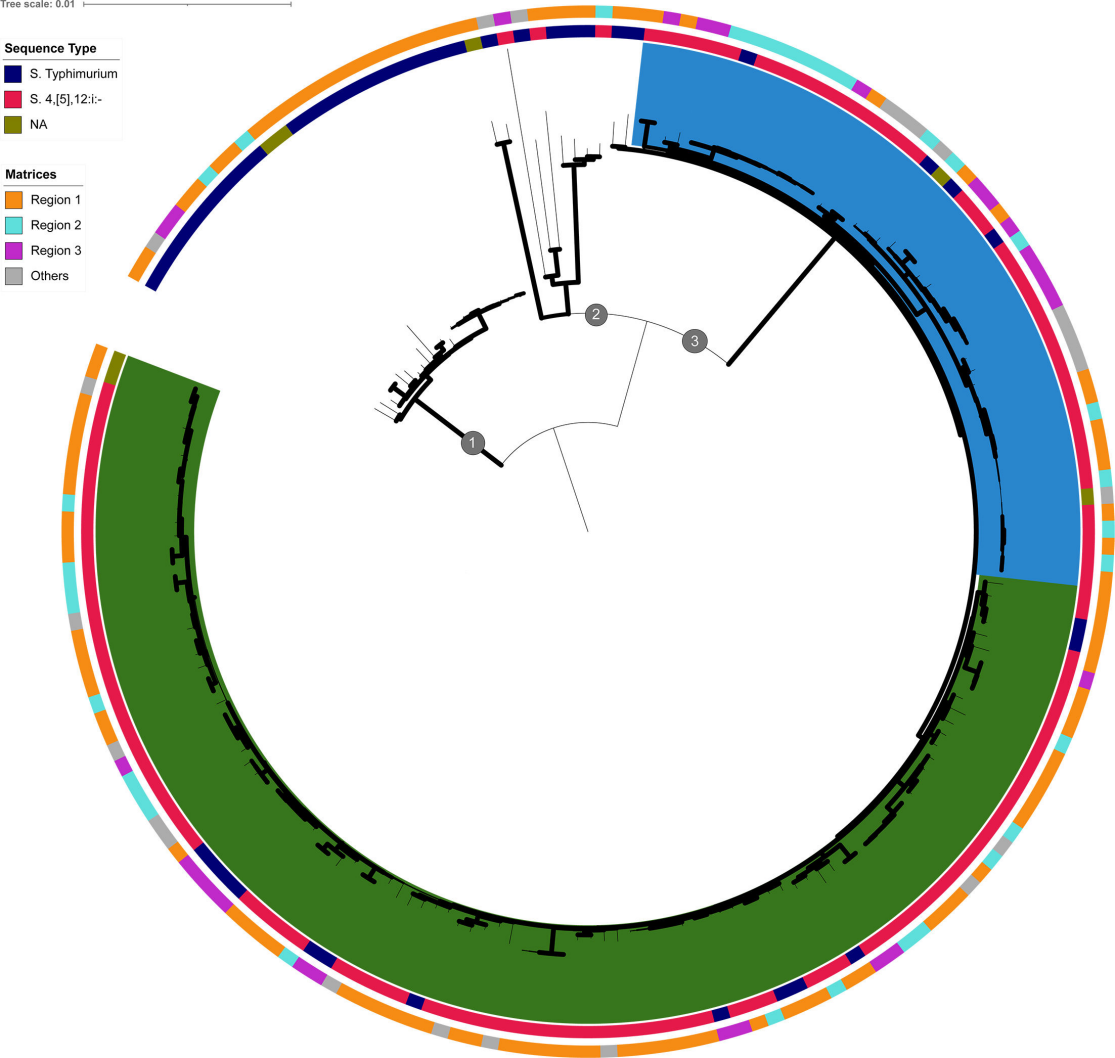
Phylogenetic reconstruction of the 188 *Salmonella* Typhimurium and 4,[5],12:i:- genomes belonging to ST 19 and ST 34 isolated in France from pig and pork production. The serovars and source of strains are indicated in the two rings around the tree. Thick black branches correspond to branches with a bootstrap of over 90%. Samples in blue or green are separated by the tree’s topology. Numbers 1, 2, and 3 correspond to the three topological clusters described in the Results section.

Among the 188 genomes selected for this study, 156 were identified *in silico* as belonging to ST 34, 24, and ST 19, and 8 were undetermined. Whole genome alignment led to the identification of 4,247 SNPs, of which 132 were identified in 25 homologous recombination events, with a total of 3,668 bp (14 in leaves and 11 in internal nodes). Excluding variants from homologous recombination, the tree was inferred by IQ-TREE following an evolutionary model of TVM + F + I, then converged with a commensurate negative likelihood (−6,787,102.637) after 102 iterations. Most of the nodes were identified with high bootstrap values (Fig. 2, in bold).

The tree is composed of three main topological clusters (nodes 1, 2, and 3 in Fig. 2). On the left (node 1, Fig. 2), one cluster is composed of 27 genomes, 25 of which are *S*. Typhimurium as identified by conventional serotyping. Two strains of this cluster were not analyzed by slide agglutination, but the *in silico* serotyping confirmed the *S*. Typhimurium prediction. Twenty of these genomes belonged to ST 19, one to ST 34, and six to an undetermined ST (Fig. S1). In this cluster, 11 samples isolated from the same pig herd were clustered together (strains in bold in Fig. S1), demonstrating little diversity of an *S*. Typhimurium clone circulating in this herd.

The second cluster (node 2, Fig. 2) comprised nine genomes with five *S*. Typhimurium, three *S*. 4,[5],12:i:-, and one strain not analyzed by conventional serotyping but attributed to the *S*. Typhimurium serovar by *in silico* genome analysis. Within this cluster, six genomes belong to ST 19, one to ST 34, and two to an undetermined ST (Fig. S1).

The third cluster (node 3 and cluster blue and green, Fig. 2) comprised 152 *S*. 4,[5],12:i:- genomes identified by conventional serotyping and five genomes attributed to the *S*. 4,[5],12:i:- by *in silico* genome analysis. All these genomes were predicted as ST 34. Overall, the third cluster was less diverse than the other two clusters, with only 34 SNP differences between genomes (Table S3, “French SNP distance” tab). Topologically, an inner node of the tree splits the 152 genomes into two sub-clusters, one with 104 and the second with 48 samples, with an intracluster mean of 49 (Fig. 2 in blue) and 51 SNPs (Fig. 2 in green), respectively. The core genome analysis could not be used to correlate the main two French sub-clusters either with sources (farm or slaughterhouse) or with geographical origin (regions 1, 2, and 3) (Fig. 2). Finally, two genome outliers (17Q002757 and 17Q002744) were observed between the three clusters.

No correlation between geographical area and phylogenetic clustering was observed here. Strains isolated from pigs from farms in the same regions were distributed all around the tree. On the contrary, several strains from different regions clustered together, for example, strains 17Q003567 isolated from a pig from region 1, 17Q003094 from region 2, and 17Q003801 from region 3 (located at the north-eastern part of the tree, Fig. S1). There was a total SNP distance between these strains of six SNPs: three SNPs between 17Q003567 and 17Q003801, and six SNPs between 17Q003094 and 17Q003801. Interestingly, these three strains were isolated by swabbing either the carcasses or tongues of pigs in two different slaughterhouses. While strain 17Q003567 was isolated in a slaughterhouse in region 1, strains 17Q003094 and 17Q003801 both came from the same slaughterhouse in region 2, but on different sampling dates.

### Genomic analysis of *S*. Typhimurium and *S*. 4,[5],12:i:- reveals an extensive arsenal for adapting to pig and pork production

#### Virulence genes

The virulence potential of the *Salmonella* isolates was evaluated through the presence of 44 virulence genes and 10 different SPIs detected in the *S*. Typhimurium and *S*. 4,[5],12:i:- data set studied.

**Fig 3:**
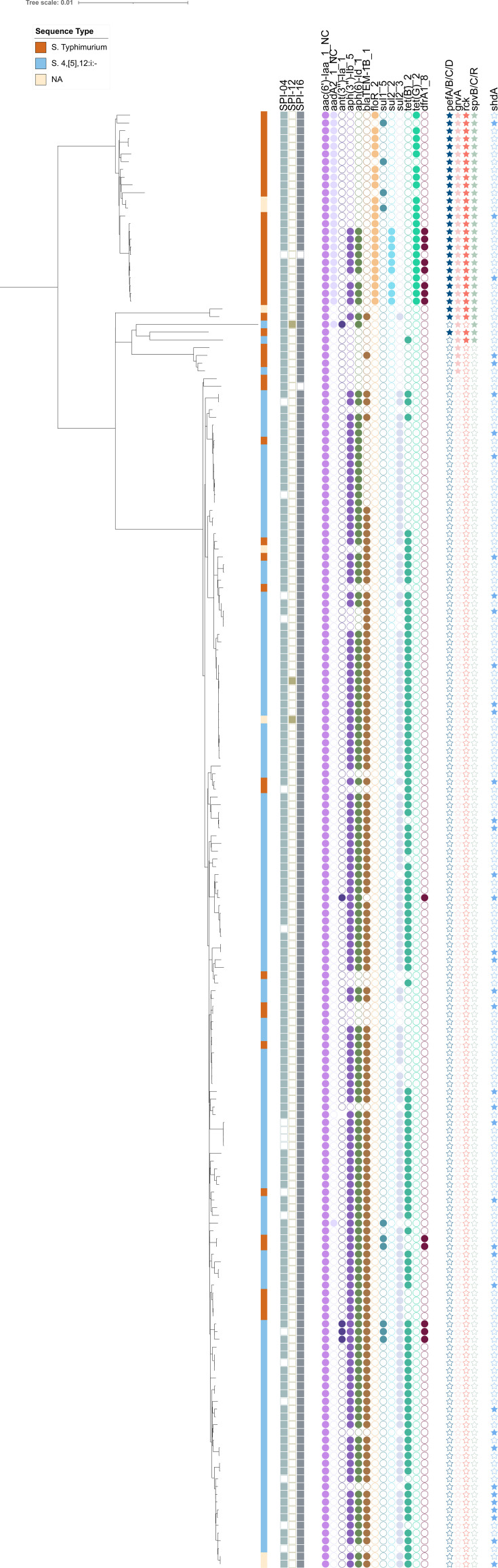
Phylogenetic reconstruction of the 188 *Salmonella* Typhimurium and 4,[5],12:i:- genomes isolated in France from pig and pork production, including the serovar, SPI, antimicrobial resistance, and virulence genes detected. Only SPI genes detected in more than five genomes are displayed. SPI and virulence genes contained in all the strains are not displayed.

All the genes coding for type III secretion systems T3SS-1 and T3SS-2, carried by SPI-1 and SPI-2, were detected in all genomes (except for virulence gene *sipD* for strain 17Q003133). SPI-1 and -2 are responsible for *Salmonella* systemic infection through penetration in non-phagocytic host cells and survival within the macrophage, respectively (38, 39). SPI-3, described as a requirement for intra-phagosomal habitat in low-Mg2+ and nutritional conditions (40), was also present in all genomes, as was SPI-5 carrying the *pipB* and *sopB* genes coding for TESS effector proteins encoded by SPI-1 and -2 (41). SPI-4, which harbors the genes responsible for adhesion to host epithelial cells and toxin secretion (42), was found in all genomes with the exception of 19, which did not match a pattern in the phylogenomic tree (Fig. 3). Further analyses would be needed to definitively assert the absence of this SPI because this pathogenic island appears to be conserved in the Typhimurium serovar.

Therefore, all the strains in the panel studied display genes conferring to serovars *S*. Typhimurium and its variant the ability to adhere to and invade the host’s epithelial cells, survive in macrophages, and establish a systemic infection. In addition, SPI-9, -13, -14, and -16 were identified in all the genomes. SPI-9, which is also found in the genomes of *Salmonella* Typhi, codes for a T1SS type secretion apparatus similar to that encoded by SPI-1 and also for proteins that allow adhesion under conditions of high osmolarity. SPI-13 and SPI-14, identified in avian-adapted *Salmonella* Gallinarum (which causes typhoid in poultry), have already been seen in *S*. Typhimurium as well as in *S*. Enteritidis, but the role of proteins encoded by these SPIs is not yet clear and requires further molecular characterization. SPI-16, previously identified in *S*. Typhi and encoding for genes that are responsible for lipopolysaccharide modifications (37, 43), was absent in only two genomes (*S*. Typhimurium BCV-16-18150-12 and 17Q002757) that were not clustered together.

Finally, SPI-12 was identified in three genomes (*S*. 4,[5],12:i:- 2014LSAL03857, 2021LSAL06139, and EmisE1_8L7), two of which are clustered together, while the third has a long branch. This SPI-12 has already been identified in *S*. Typhimurium and *Salmonella* Cholerasuis, a host-adapted pathogen that causes swine paratyphoid (44). SPI-12 encodes a remanent phage known to contribute to bacterial virulence, but the fact that this SPI was rare in our data set showed that it is not needed for *Salmonella* Typhimurium and 4,[5],12:i:- invasion and pathogenicity.

Beyond SPIs, other virulence genes were identified. As detected previously with other *Salmonella* serovars, gene clusters of *csg* (45), *fim* (46), *shdA*, *bcf* (47), and *lpf* involved in the biogenesis of Curli fibers and fimbriae have also been identified in all the genomes. Interestingly, some of these genes play a role in the regulation of biofilm formation when *Salmonella* colonizes the intestines (48). Additionally, the *rat*B effector gene coding for a secreted protein associated with intestinal colonization and persistence was identified in all the genomes (49). This gene has also been identified in other serovars and strains isolated from pigs (50). The *pef* and *grvA* genes, coding for fimbriae and antivirulence, as well as the *spv* and *rck* genes, described as part of ancestral virulence plasmids, were identified only in *S*. Typhimurium samples (Table S2, “VFDB” tab and Fig. 3). Further analysis would be needed to identify the presence of IncFIB and IncFII virulence plasmids within the *S*. Typhimurium isolates analyzed.

#### Antimicrobial resistance genes

The antimicrobial resistance (AMR) genes detected are illustrated in Fig. 3; Table S2, “Resfinder” tab. ST 34 is known to harbor multiple AMR genes related to the tetracycline, ampicillin, sulfisoxazole, and streptomycin antibiotics (51). In our data set, AMR genes against aminoglycoside (*aph*, *aac*), beta-lactam (*blaTEM-1B_1*), phenicol (*floR_2*), sulfonamide (*sul*), tetracycline (*tet*), and trimethoprim (*dfr*) were detected. We observed AMR differences between ST 19 and ST 34 strains: beta-lactam resistance was carried by most ST 34 strains (80% of ST 34 and 16% of ST 19), whereas resistance to phenicol was carried by ST 19 strains only (Table S2, “Resfinder” tab) through the *floR* gene (2% of ST 34 and 55% of ST 19).

Looking at Fig. 3, some strains shared the same resistance but did not carry the same genes: sulfonamide and tetracycline resistance is carried by *sul1_5*, *sul2_2,* and *tet(G)_2* for the ST 19 cluster, while the same resistance is carried by *sul_2_3* and *tet(B)_2* for the ST 34 strains. Finally, it is interesting to note that some ST 19 strains belonging to cluster 1 have an additional aminoglycoside gene (*aad*), which is not present in the ST 34 strains of clusters 2 and 3. No difference was observed between the two ST 34 strain sub-clusters.

### Worldwide phylogenomic analysis of *S*. 4,[5],12:i:- ST34

As the core genome diversity of the cluster characterized by ST 34 in the French pig sector seems to split into only two sub-clusters, a worldwide analysis was carried out to understand whether this diversity was shared by other ST 34 strains isolated from different countries. Within the 188 French genomes analyzed in this study, 132 ST 34 genomes were selected for this analysis. These genomes were chosen because they did not cluster with any ST 19 or *S*. Typhimurium strains in the French phylogenomic tree (Fig. 2). We added 193 genomes collected from Enterobase from foreign countries, thus reaching a total of 325 genomes in the data set. A phylogenomic tree excluding homologous recombination variants (114 SNPs) was inferred, following an evolutionary model of TVM + F + I, and converged with a commensurate negative likelihood (−6,795,270.421) and high bootstrap values (Fig. S2) after 140 iterations. The mean SNP difference between all samples from the tree was 68 SNPs (Table S3, “World SNP distance” tab). This is similar to the ST 34 French tree diversity, suggesting worldwide dissemination of the same ST 34 clone.

**Fig 4:**
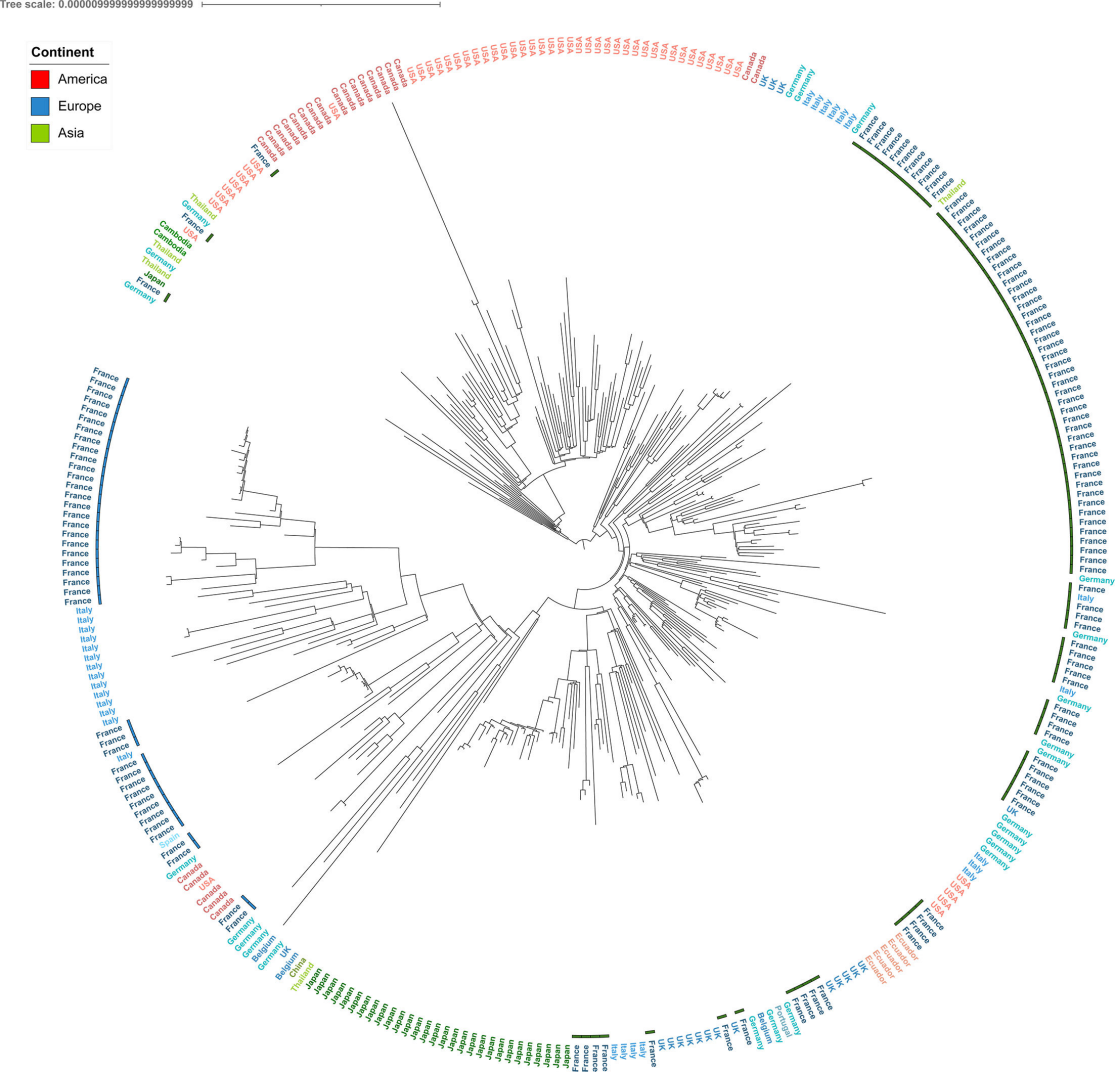
Phylogenetic reconstruction of the 325 ST 34 genomes isolated in France and other countries from pig and pork production. The annotation corresponds to the strain’s country of isolation, colored by continent. Samples are shown in blue or green according to their topological position in Fig. 2.

Overall, French genomes appeared to cluster into two main groups (Fig. 4), confirming the circulation of two clones, with some exceptions concerning isolates from bordering countries, which could be explained by the spread of ST 34 throughout all European countries due to trading, as previously observed by Cadel-Six et al. (12) in 2021.

The French genomes belonging to sub-cluster 1 of the French tree (blue group in Fig. 4) were grouped with 14 genomes from Italy, 1 from Spain, and 1 from Germany (Fig. S2). Two French genomes (17Q003798 and 17Q003795) from sub-cluster 1 seemed to group with eight North American genomes (five from Canada and one from the United States), but if we refer to the internal node, a significant genetic distance exists between these genomes (Fig. S1). Moreover, these two genomes were further away from the rest of the sub-cluster, which could explain this topological position in the international tree.

Of the 91 (89%) French samples belonging to sub-cluster 2 (green group in Fig. 4), 81 samples were grouped with 33 European samples (11 from Italy, 13 from Germany, and 9 from the United Kingdom), 10 American samples (five from the USA and five from Ecuador), and 1 from Thailand. Among these samples, two German samples isolated from pork were quite close to French strains, which were collected from four different slaughterhouses not located in the same region. This proximity could be explained by the trading of piglets or raw meat between these two bordering countries. For the other strains, it was not possible to make assumptions about their link because of the lack of epidemiological information.

For the remaining 10 French genomes in sub-cluster 2, 7 shared the same nodes with 29 Japanese and 17 European genomes (four from Italy, eight from the UK, three from Germany, one from Belgium, and one from Portugal). Out of these seven French samples, four were singletons with long branch distances, while the other three were clustered together with a short SNP distance (a mean of three SNPs). Finally, one sample (17Q003076) grouped into a cluster within American samples from the USA (*n* = 45) and Canada (*n* = 18). This French genome was sampled from a pig carcass at the slaughterhouse and differed from the USA strain (SRR5801109) by only 15 SNPs, whereas it had many SNPs (at least 49) in common with other French strains. The last two French samples each grouped with two different German strains into a cluster supported by a low bootstrap value (59) with genomes from Cambodia (*n* = 2), Germany (*n* = 1), Japan (*n* = 1) Thailand (*n* = 2), and the USA (*n* = 1).

## DISCUSSION

### Diversity of *S*. Typhimurium and *S*. 4,[5],12:i:- strains in France

In this study, the diversity of *S*. Typhimurium was observed to be higher than that of *S*. 4,[5],12:i:- in the pig and pork sector in France, with the dissemination of probably one to two clones of *S*. 4,[5],12:i:- on all French pig farms. The greater genetic diversity of serovar Typhimurium than its monophasic variant observed in this study has already been reported in the literature, as previous studies have described *S*. 4,[5],12:i:- genomes as less heterogeneous than *S*. Typhimurium ones (52, 53). As Tassinari et al. (54) suggested in 2019, it seems logical to think that the monophasic variant ST 34, derived from serovar Typhimurium around the 1980s and having recently colonized the pig sector, has undergone a limited sequence divergence (54).

For *S*. Typhimurium and *S*. 4,[5],12:i:- strains, clonal patterns suggest either contamination of multiple farms from a common source or direct transmission between farms in regions 1, 2, and 3. Feed has also been suggested as a possible common source of contamination (54, 55). Previous studies observed little divergence between strains from different farms based on SNP distance and multilocus variable number tandem repeat analysis, and in some cases, identical or near-identical strains were also isolated from feed mills, suggesting a common source of contamination through feed (56, 57). This source could explain here how strains differing by only three or six SNPs can be found on farms geographically distant, but this hypothesis must be treated with caution. Further studies with more feed samples should be carried out to prove or disprove this hypothesis. On the other hand, *Salmonella* cross-contamination between carcasses has also been documented in slaughterhouses (58). Recycled water used in the dehairing machine has been identified in the literature as an important source of *Salmonella* contamination through the slaughter line (59). Water at slaughterhouses could thus be the source of contamination and explain why similar strains are isolated from carcasses of animals from different farms. However, while most of the samples in this study were taken from carcasses at the slaughterhouse (167/188), in France, pigs from different farms are kept in separate rooms at the slaughterhouse in order not to mix the animals and thus limit cross-contamination between animals/carcasses from different farms. Moreover, most of the pig farms are located quite near a slaughterhouse, with a mean travel distance of 120 km (22). In this study, while these data should not change significantly for regions 1 and 3, slaughterhouses in region 2 may receive pigs from region 1. In this case, cross-contamination could occur between animals from farms in regions 1 and 2, but this does not explain cross-contamination between pigs from farms in regions 1 and 3. Finally, data such as information regarding the movement of animals and all risks of contamination related to these movements (feed, livestock exchanges, contamination of the transport vehicle, cross-contamination during transport from farms to the slaughterhouse, or trade agreements between producer groups and slaughterhouses not necessarily in the same region) would be needed to fully grasp the whole picture.

In this study, the phylogenetic results interpreted with regard to the location of the pig-breeding origin of the samples revealed that two different clones of *S*. 4,[5],12:i:- are probably circulating on French pig farms. These two clones are distributed evenly throughout the three French regions with the highest number of pig farms in France (Brittany, Pays-de-la-Loire, and Nouvelle-Aquitaine) as well as in the seven other regions analyzed. These results underline the importance of carrying out sampling campaigns directly on farms. Indeed, on-farm studies are unfortunately rare because conducting sampling campaigns on farms in 10 different French regions is both challenging and very expensive, so most studies are based on samples collected at the slaughterhouse during national monitoring plans mandated by the state. To elucidate the on-farm situation, DGAL changed the sampling protocol at slaughterhouses in 2021: now the official sample collections mandated by the state are carried out directly on the feces of the animals upon arrival at the slaughterhouse. Our study is, therefore, located upstream of this change in French regulations and is pioneering in its results on the situation of pig farms in France. Finally, we are currently constituting an extensive collection of 350 strains from the pig industry, covering breeding to finished products, and including strains from human cases that occurred between 2019 and 2023 following the ingestion of pork. This collection will be used to reinforce our genomic and phenotypical knowledge of the clones of S. 4,[5],12:i:- circulating in French pig and pork production.

### French ST 34 diversity compared with its worldwide diversity

In this study, even if global ST 34 data may present certain biases (e.g., the strains from the USA were selected on the basis of the metadata available and not of their geographical diversity, and the Asian data set is biased by the high number of Japanese strains), the two French topological sub-clusters are unaffected. Most of the ST 34 strains belonging to the two French sub-clusters are distributed between two of the five main groups identified by international phylogenetic analysis, with some French strains disseminated along the tree within the other groups. Most French strains clustered with European strains isolated from Italy and Germany. This is consistent with trading between these European Union countries, which represented 22% of the pigs exported from France worldwide in 2021 (60). On the other hand, the French strains that were associated with the USA or Japanese ones could be explained by the inter-continental traveling of infected or convalescent persons, whether tourists or pig sector workers. The hypothesis of contaminated feed, as described above, should not be excluded either. Finally, the direct international trading of live animals could also explain this phenomenon. Indeed, as observed previously with *S*. Enteritidis in poultry strains (61), in a world of globalized markets, the international trading of animals is increasingly common. A phylodynamic and spatiotemporal characterization of world strains in pig and pork production could highlight the driving role of trade in the dissemination of ST 34, especially for groups of strains with little diversity. The international transmission of *S*. Typhimurium has already been highlighted in humans (62–64), but focused on AMR genes or mobile elements, without metadata on the contaminated food. Adding this kind of information to this study could bring a new vision of global transmission, especially in an era of global food epidemics (65, 66). Interestingly, it has also been shown that ST 34 was introduced into the United States from Europe on multiple occasions, notably in the pork industry (67). This link between Europe and the USA (68) was established by time-scaled phylogenetic analysis of isolates and a focus on the presence of plasmid-mediated resistance genes, which would be interesting to analyze on a French level to understand whether sub-clusters could be the product of several phases in the emergence of ST 34. Other sampling matrices could be considered to better understand ST 34 in the global pig and pork production sector. It has been previously demonstrated that the environment (69) could explain some *Salmonella* clusters. A study in Japan demonstrated that genomic clades for *S*. Typhimurium and *S*. 4,[5],12:i:- strains were mixed between different animal sources, with some pig strains clustering with cattle strains (70). It also pointed out that the predominant clone’s characteristics were similar to European clones, which could explain why some French strains are very close to the clade of the Japanese strain in our data.

### Persistence of strains (AMR SPI)

In this study, we showed that *S*. Typhimurium and *S*. 4,[5],12:i:- strains isolated from pigs carry several virulence and resistance genes that may explain their persistence in pig and pork production facilities. Several virulence genes were identified, but their expression should be further analyzed with suitable phenotypical methods. For example, *grvA* was described as an antivirulence gene because it has been demonstrated that this gene induces a decrease in *S*. Typhimurium virulence among mice (71). This gene is only present in *S*. Typhimurium strains, which could mean that *S*. 4,[5],12:i:- may be more virulent due to its absence.

Regarding virulence factors, SPIs previously observed in *S*. Typhimurium or *S*. 4,[5],12:i:- strains were identified in the French data set. These include SPI-1, SPI-2, SPI-3, SPI-4, SPI-12, and SPI-16 (39, 40, 44, 72–74). However, in some data sets on pigs from other countries, we observed that SPI diversity may differ. For example, in Brazil, SPI-1, SPI-2, SPI-3, SPI-4, SPI-5, SPI-8 (in 2.6% of the isolates), SPI-9, SPI-13, and SPI-14 have been identified (75). In the literature, SPI-5 has been described less frequently in *S*. Typhimurium, but more in *Salmonella* Newport and *S*. Enteritidis (76, 77), as it encodes effectors of SPI-1 and SPI-2 that displayed virulence in these serovars. SPI-13 is not needed to internalize *Salmonella* among humans, but it is for some other animals, such as mice (78). Therefore, its presence or absence in pigs may reflect the pathogen’s introduction into the herds.

The presence of SPI-14 has been observed in some *S*. Typhimurium strains and contributes to virulence by influencing invasion through the *loiA* virulence gene, which activates the transcription of *hilD*, leading to the activation of *hilA* (the main regulator of SPI-1) (79, 80). This SPI has been mostly studied in other serovars, such as *S*. Enteritidis (81) and *S*. Gallinarum (82) in poultry matrices. However, comparison between these studies remains difficult because the parameters or tools used to determine the presence of SPIs differ from one study to another.

One main difference detected in this data set is the beta-lactam resistance carried by most of the *S*. 4,[5],12:i:- strains, whereas resistance to phenicol was carried by *S*. Typhimurium strains only (Table S2, “Resfinder” tab). Phenicol resistance has already been identified in *S*. Typhimurium (83, 84), and beta-lactam resistance associated with *S*. 4,[5],12:i:- isolates (51) has also been observed. It is also interesting to note that beta-lactam resistance has been identified in some *S*. Typhimurium strains, but seems to be predominant in *S*. 4,[5],12:i:- strains (12). However, most of the virulence and resistance genes identified were present in all strains, whatever the source. This observation may indicate that strains have developed an extensive arsenal of resistance and virulence genes that allow them to survive throughout the pig and pork production sector. Nevertheless, this could also be the case in other food sectors (85, 86).

Mobile elements play a very important role in the acquisition and transmission of AMR genes. *aadA2_1_NC*, *tet(G)_2*, *aac(6′)-Iaa_1_NC,* and *floR_2*, genes mediating resistance to aminoglycosides, tetracyclines, beta-lactams, and florfenicol, can be associated with an integron region of *Salmonella* Genomic Island 1 (12). *aph(3″)-Ib_5*, *aph(6)-Ib_1*, *blaTEM-1B_1*, *sul2_3,* and *tetB_2* are also associated with the presence of *Salmonella* Genomic Island 4 in the strains (12, 87).

Other AMR genes detected here could be carried by different plasmids, such as the *blaTEM-1B* gene that has already been identified on the IncF family plasmid in *Salmonella* strains (88, 89). The *sul1* and *sul2* genes are the most frequent sulfonamide resistance genes present in *Salmonella* and are associated with plasmids and transposons (90). Finally, the *dfrA1* gene has been identified in a class 1 integron in the *S*. Typhimurium strain (91). These mobile elements could explain the diversity of AMR strains in our data set and even in the sub-clusters identified.

As discussed previously, *S*. 4,[5],12:i:- strains are circulating in Europe and are present in all sectors from the farm and its environment to humans. However, they remain predominantly associated with the pig reservoir and do not persist for long at individual farm level in other species such as cattle (92). Further analysis comparing *S*. Typhimurium and *S*. 4,[5],12:i:- genomes from different animal hosts could highlight resistance genes present only in pig and pork strains, potentially explaining their persistence in this host. In addition, the possibility of geographical segregation linked to the presence of phages has already been demonstrated (93) but needs to be verified in order to determine whether or not the genomic diversity identified in this study is associated with this type of accessory content.

## Data Availability

Raw reads of the project have been deposited in the SRA under accession number PRJNA1051311.

## SUPPLEMENTAL MATERIAL

Supplemental figures spectrum.00526-24-s0001.pdf Fig. S1 and S2.

Table S1 spectrum.00526-24-s0002.xlsx Epidata of strains selected from France and from others countries for this study.

Table S2 spectrum.00526-24-s0003.xlsx Results from SPIFinder, VFDB and ResFinder databases for French genomes.

Table S3 spectrum.00526-24-s0004.xlsx SNP pairwise matrix distance for the French phylogenetic tree and the worldwide phylogenetic tree as calculated by GATK (iVARCall2 pipeline).

ASM does not own the copyrights to Supplemental Material that may be linked to, or accessed through, an article. The authors have granted ASM a non-exclusive, world-wide license to publish the Supplemental Material files. Please contact the corresponding author directly for reuse.
