# Supplementary material for: Genomic diversity of Salmonella Typhimurium and its monophasic variant in pig and pork production in France
Source: Microbiol Spectr. 2024 Nov 8;12(12):e00526-24. doi: 10.1128/spectrum.00526-24 (PMC11619346; doi:10.1128/spectrum.00526-24)
Supplement: Supplemental figures — Fig. S1 and S2. [file spectrum.00526-24-s0001.pdf]

Supplementary Material

Supplementary Figure 1: Phylogenetic reconstruction of the 188 *Salmonella* Typhimurium and 4,[5],12:i:- genomes found in French pig and pork production. Samples are coloured by ST. If the sample name is in bold, it was isolated on a a pig farm.

Supplementary Figure 2: Phylogenetic reconstruction of the 325 ST34 genomes isolated in France and other countries from pig and pork production. Bootstrap numbers > 90% are annotated on the branches.

Supplementary Table 1: Epidata of strains selected from France and other countries for this study.

Supplementary Table 2: Results from SPIFinder, VFDB and ResFinder databases for French genomes. The presence or absence of the gene or genomic region were obtained using a threshold set at 90% identity over at least 3/5 of the sequence length.

Supplementary Table 3: SNP pairwise matrix distance for the French phylogenetic tree and the worldwide phylogenetic tree as calculated by GATK (iVARCall2 pipeline).

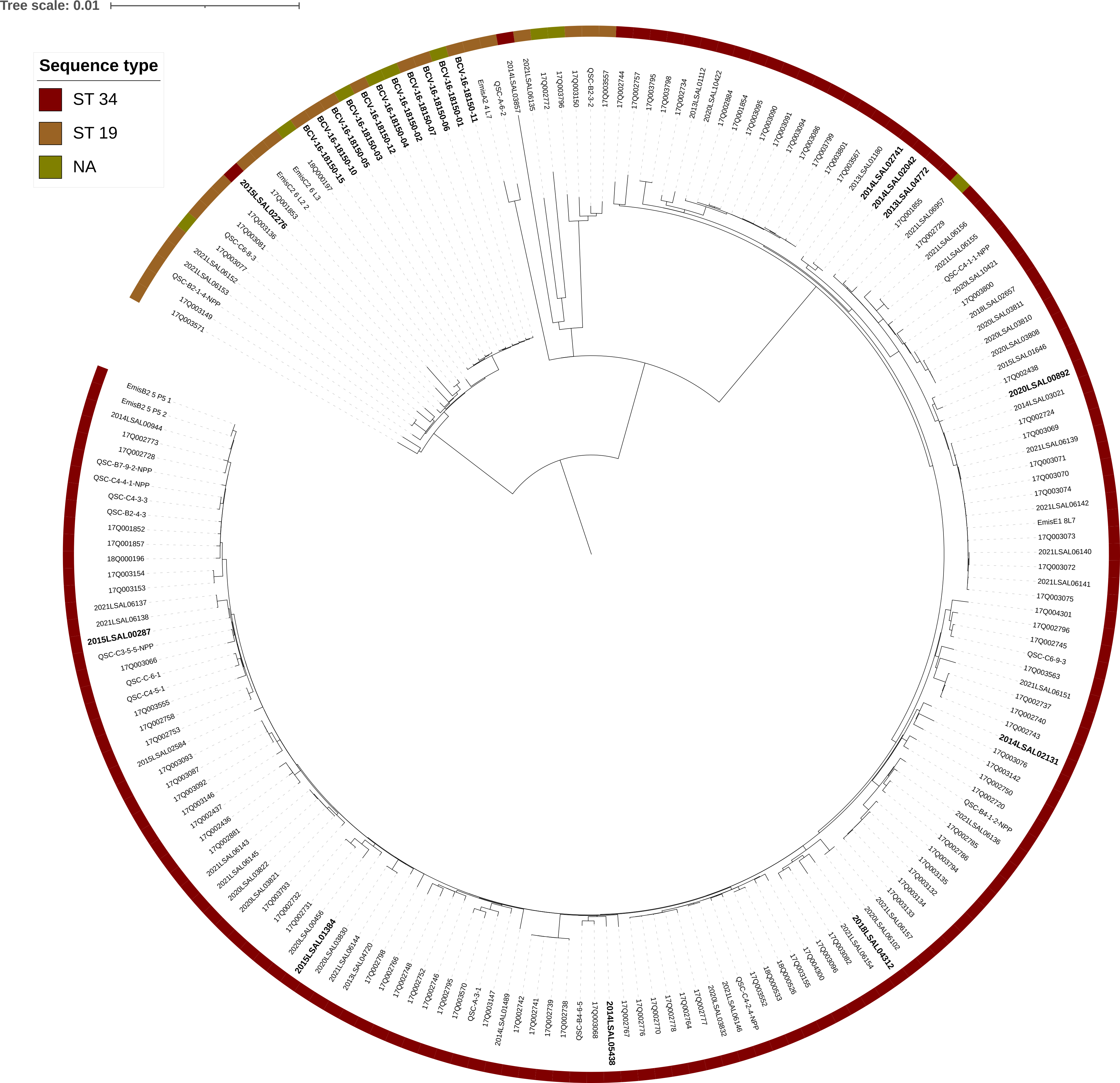
Tree scale: 0.01
Sequence type
ST 34
ST 19
NA
17Q003571
17Q003149
QSC-B2-1-4-NPP
2021LSAL06153
2021LSAL06152
17Q003077
QSC-C6-8-3
17Q003081
17Q003136
2015LSAL02276
17Q001853
EmisC2 6 L2 2
EmisC2 6 L3
18Q000197
BCV-16-18150-15
BCV-16-18150-10
BCV-16-18150-05
BCV-16-18150-03
BCV-16-18150-12
BCV-16-18150-04
BCV-16-18150-02
BCV-16-18150-07
BCV-16-18150-06
BCV-16-18150-01
BCV-16-18150-11
EmisA2 4 L7
QSC-A-6-2
2014LSAL03857
2021LSAL06135
17Q002772
17Q003796
17Q003150
QSC-B2-3-2
17Q003557
17Q002744
17Q002757
17Q003795
17Q003798
17Q002734
2013LSAL01112
2020LSAL10422
17Q002884
17Q001854
17Q003095
17Q003090
17Q003091
17Q003094
17Q003086
17Q003799
17Q003801
17Q003567
2013LSAL01180
2014LSAL02741
2014LSAL02042
2013LSAL04772
17Q001855
2021LSAL06957
17Q002729
2021LSAL06156
2021LSAL06155
QSC-C4-1-1-NPP
2020LSAL10421
17Q003800
2018LSAL02657
2020LSAL03811
2020LSAL03810
2020LSAL03808
2015LSAL01646
17Q002438
2020LSAL00892
2014LSAL03021
17Q002724
17Q003069
2021LSAL06139
17Q003071
17Q003070
17Q003074
2021LSAL06142
EmisE1 8L7
17Q003073
2021LSAL06140
17Q003072
2021LSAL06141
17Q003075
17Q004301
17Q002796
17Q002745
QSC-C6-9-3
17Q003563
2021LSAL06151
17Q002737
17Q002740
17Q002743
2014LSAL02131
17Q003076
17Q003142
17Q002750
17Q002720
QSC-B4-1-2-NPP
2021LSAL06136
17Q002785
17Q002786
17Q003794
17Q003135
17Q003132
17Q003134
17Q003133
2021LSAL06157
2020LSAL06102
2018LSAL04312
2021LSAL06154
17Q003082
17Q003096
17Q004300
17Q003155
18Q000526
18Q000533
17Q003552
QSC-C4-2-4-NPP
2021LSAL06146
2020LSAL03832
17Q002777
17Q002764
17Q002778
17Q002770
17Q002776
17Q002767
2014LSAL05438
17Q003068
QSC-B4-6-5
17Q002738
17Q002739
17Q002741
17Q002742
2014LSAL01489
17Q003147
QSC-A-3-1
17Q003570
17Q002795
17Q002746
17Q002752
17Q002748
17Q002766
17Q002798
2013LSAL04720
2021LSAL06144
2020LSAL03830
2015LSAL01384
2020LSAL00456
17Q002731
17Q002732
17Q003793
2020LSAL03821
2020LSAL03822
2021LSAL06145
2021LSAL06143
17Q002881
17Q002436
17Q002437
17Q003146
17Q003092
17Q003087
17Q003093
2015LSAL02584
17Q002753
17Q002758
17Q003555
QSC-C4-5-1
QSC-C-6-1
17Q003066
QSC-C3-5-5-NPP
2015LSAL00287
2021LSAL06138
2021LSAL06137
17Q003153
17Q003154
18Q000196
17Q001857
17Q001852
QSC-B2-4-3
QSC-C4-3-3
QSC-C4-4-1-NPP
QSC-B7-9-2-NPP
17Q002728
17Q002773
2014LSAL00944
EmisB2 5 P5 2
EmisB2 5 P5 1

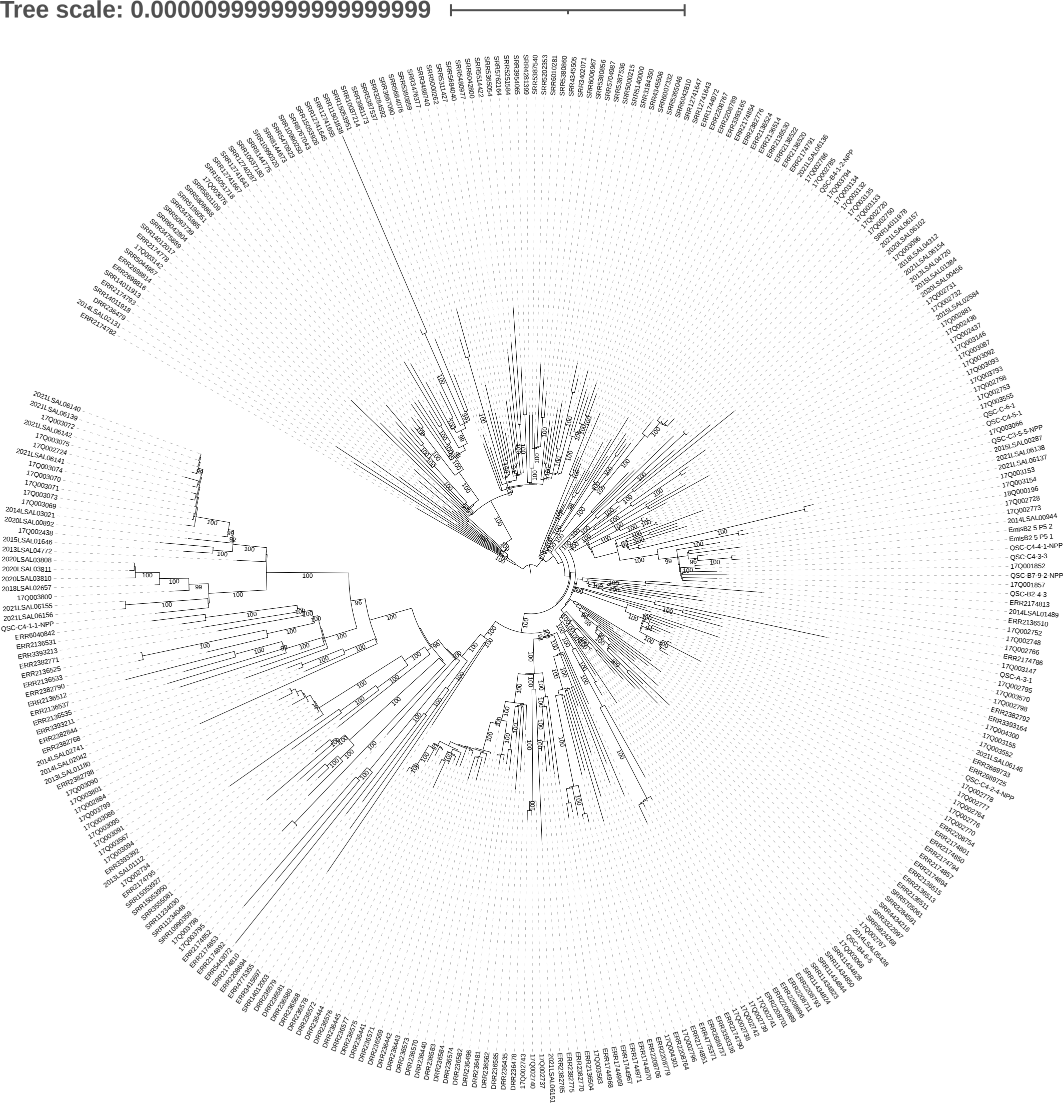
Tree scale: 0.0000009999999999999999
